# Protective Effects of Polysaccharides From *Sargassum hemiphyllum* (Turner) C. Agardh Against Alcohol‐Induced LO2 Cell Damage

**DOI:** 10.1002/fsn3.4632

**Published:** 2024-12-01

**Authors:** Yuxuan Liang, Zhuo Wang, Rui Li, Saiyi Zhong, Xiaofei Liu, Jianping Chen

**Affiliations:** ^1^ College of Food Science and Technology Guangdong Ocean University, Guangdong Provincial Key Laboratory of Aquatic Product Processing and Safety, Guangdong Provincial Engineering Technology Research Center of Seafood, Guangdong Province Engineering Laboratory for Marine Biological Products, Key Laboratory of Advanced Processing of Aquatic Product of Guangdong Higher Education Institution Zhanjiang China

## Abstract

The study aimed to explore the protective impact of polysaccharide derived from *Sargassum hemiphyllum* (Turner) C. Agardh (SHP) against ethanol‐induced injury in LO2 hepatocytes, along with its potential mechanism of action. A model of alcoholic injury in LO2 cells was established to assess the shielding effect of SHP against liver injury induced by alcohol. Treatment with 800 mmol/L ethanol for 6 h was selected for the construction of the hepatocyte injury model. Compared with those in the alcohol model group, the survival rate of LO2 cells in the SHP treatment group was significantly greater. When the concentration of SHP reached 60 μg/mL, the cell viability increased to 89.17% ± 3.58%. Moreover, SHP treatment significantly reduced the level of intracellular reactive oxygen species (ROS), increased the levels of intracellular glutathione (GSH), lactate dehydrogenase (LDH), and catalase (CAT), reduced the level of malondialdehyde (MDA), and prevented the leakage of intrahepatic enzymes (aspartate aminotransferase (AST) and alanine transaminase (ALT)) to protect LO2 cells from alcohol‐induced injury. Moreover, at a concentration of 60 μg/mL, SHP inhibited the ethanol‐induced reduction in the protein expressions of Nrf2, HO‐2, and GCLC, indicating its potential to modulate the antioxidant system to restore the homeostatic state, consequently shielding the liver from peroxidative damage induced by alcohol. These results propose that SHP exhibits a protective role against oxidative damage in LO2 cells and holds promise as a novel natural hepatoprotective agent for averting liver injury.

## Graphical abstract

We investigated the protective effect of *Sargassum hemiphyllum* (Turner) C. Agardh polysaccharide (SHP) against ethanol‐induced LO2 cell injury. It was found that *Sargassum hemiphyllum* (Turner) C. Agardh polysaccharide (SHP) could protect against ethanol‐induced liver injury. Further mechanistic studies showed that SHP treatment inhibited cellular oxidative stress by upregulating the levels of Nrf2 and HO‐1, thereby attenuating liver injury.

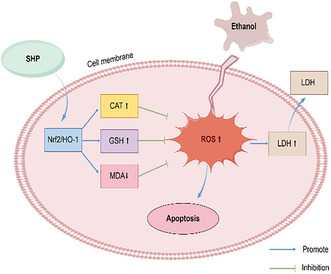

## 1 Introduction

In recent years, the prevalence of health issues stemming from alcohol consumption and abuse has risen, leading to an annual increase in alcoholic liver disease (ALD) cases. Globally, ALD accounts for 3.8% of yearly fatalities (Marroni et al. 2018). The liver, being the primary metabolic organ, is particularly vulnerable to alcohol‐induced toxicity, metabolizing over 90% of ingested alcohol. ALD, a consequence of excessive alcohol intake, typically initiates with fatty liver symptoms and can progress to alcoholic hepatitis, liver fibrosis, cirrhosis, and even liver cancer, posing significant health and societal challenges (Su et al. 2023). Presently, the primary treatment approach for ALD involves alcohol cessation, nutritional supplementation, management of disease severity, addressing secondary malnutrition, and symptomatic treatment of alcoholic cirrhosis and its complications (Mathurin et al. 2012). Despite advancements in modern medicine, there is still a lack of specific curative drugs for liver diseases, with conventional anti‐alcoholic medications often carrying side effects or introducing new toxicity issues. Consequently, there is a growing interest in developing natural, low‐toxicity hepatoprotective agents to counter alcoholic liver injury, a shared concern within medical and food science disciplines that holds considerable research importance.

Brown algae, as the second‐largest population of marine algae, are recognized for their significant research value owing to biodiversity. China possesses abundant brown algae resources, with *Sargassum hemiphyllum* (Turner) C. Agardh being the most prevalent species, thriving notably in Salammoniac Island of Zhanjiang, Guangdong Province. Among its vital constituents, polysaccharide stands out. It is documented that the polysaccharide of *Sargassum hemiphyllum* (Turner) C. Agardh encompasses sulfuric acid groups, constituting a distinctive active ingredient commonly referred to as fucoidan. Fucoidan, a natural macromolecular polysaccharide predominantly present in brown algae, exhibits notable antioxidant, antitumor, immunomodulatory, and ethanol dehydrogenase activation activities, making it a subject of increasing research interest (Ganesan, Tiwari, and Rajauria 2019; Healy et al. 2023). Guided by contemporary medical theory, studies on fucoidan and its mechanisms in oxidative stress, glycolipid metabolism, inflammatory response, tumor proliferation, and metastasis have emerged as a novel research avenue, offering insights into potential hepatoprotective interventions (Li, Guo, and Wu 2020). Consequently, it is postulated that sargassum polysaccharides hold promise in promoting alcohol metabolism and safeguarding against alcoholic liver injury in vivo. Nevertheless, scant attention has been given to the cellular‐level protective effects of Sargassum polysaccharides against alcoholic liver injury.

In this investigation, we developed a model of alcoholic injury in LO2 cells and examined the protective effects of SHP against alcohol‐induced damage in LO2 cells. This examination included assessing survival rates, analyzing alcohol‐metabolizing enzyme activity, and evaluating oxidative stress levels. Furthermore, we employed western blotting to explore potential protective mechanisms. Our results contribute to the theoretical groundwork for utilizing SHP clinically in the treatment of alcoholic liver injury.

## 2 Materials and Methods

### 2.1 Materials and Reagents

The polysaccharides of *Sargassum* *hemiphyllum* (Turner) C. Agardh were synthesized in the laboratory, washed, dried, powdered, and sieved, and then stored at 4°C in the dark. Glutathione tablets were obtained from Chongqing YaoPharma Co. (Chongqing, China). Anhydrous ethanol was obtained from Xilong Scientific Co. (Shantou, Guangdong, China). The cell lysate RAPI, BCA protein test kit, Hoechst 33342/PI double staining kit and reactive oxygen species detection kit were acquired from Beyotime Biotechnology Co. Ltd (Shanghai, China). The aspartate aminotransferase test kit, alanine aminotransferase test kit, lactate dehydrogenase test kit, malondialdehyde test kit, and glutathione peroxidase test kit were obtained from Nanjing Jiancheng Bioengineering Institute. Catalase assay kit was acquired from Suzhou Grace Biological Co. Fetal bovine serum, DMEM high glucose medium, 0.25% trypsin solution, and double antibody solution were purchased from Gibco (USA). Dimethyl sulfoxide (DMSO) solution was obtained from Shanghai YiEn Chemical Technology Co. Methylthiazolyldiphenyl‐tetrazolium bromide (MTT) was purchased from Shanghai Yuanye Bio‐Technology Co. Ltd. (Shanghai, China). AO/EB assay kit was purchased from Beijing Leagene Biotechnology Co. Ltd. (Beijing, China). The antibody against β‐actin was from Santa Cruz Biotechnology (Dallas, TX, USA). The antibodies against Nrf2 and HO‐1 were from Cell Signaling Technology (Beverly, MA, USA). The antibody against GCLC was from Proteintech Group (Wuhan, China). All other reagents were analytical grade.

### 2.2 Preparation of Polysaccharides From *Sargassum hemiphyllum* (Turner) C. Agardh Polysaccharide

According to the method outlined by Luo et al. (Luo et al. 2022), *Sargassum* underwent crushing and sieving (with a pore size of 0.18 mm), followed by preparation into an aqueous solution at a ratio of 1:30 (material mass: liquid volume). The solution was then subjected to extraction in a water bath at 80°C for 3.5 h. Afterward, the extract underwent ultrasonication (at 60°C, 350 W) for 50 min, centrifugation (at 4000 r/min) for 20 min, and subsequent concentration to obtain the sample concentrate. The resulting precipitate was washed alternately with acetone and anhydrous ethanol twice. Following dissolution in a small amount of water, the Sevage reagent [V (n‐butanol): V (chloroform) = 1:4] was added at a volume ratio of 5:1 (polysaccharide solution: Sevage reagent) and vigorously oscillated for 1 h. The precipitate was separated by static stratification in the separatory funnel, and this Sevage process was repeated 6 times until no denatured proteins were present. SHP was then obtained through 48 h of dialysis and vacuum freeze‐drying (Shanghai Yihang Science Co., China). The basic chemical composition of SHP was as follows: polysaccharide content 75.33% ± 3.99%, fucose content 15.63% ± 3.37%, sulfate content 29.16% ± 0.86%, and glucuronic acid content 10.46% ± 0.77%, respectively. Further infrared spectroscopic analysis showed that SHP was a sulfated polysaccharide dominated by α‐glucoside bonds with a small amount of β‐glucoside bonds, and its sulfuric acid group was attached to the sugar C2 or C3 positions (Luo et al. 2022).

### 2.3 Cell Culture and Drug Treatment

LO2 cells were obtained from ATCC, USA. LO2 cells were revived and transferred to high‐glucose DMEM (with 10% fetal bovine serum and 1% double antibody solution). The cell culture flasks were then placed in an incubator at 37°C with 5% CO_2_. Passaging was performed when the cell density reached around 80%, and cells in the logarithmic growth phase were selected for subsequent experiments. Both SHP and the positive control drug glutathione (GSH) were dissolved in basal medium through periplasmic treatment (using a 0.45 μm membrane) and stored in a refrigerator at 4°C.

### 2.4 Establishment of an Ethanol‐Induced LO2 Cell Injury Model

LO2 cells (8 × 10^4^ cells/mL) were divided into either the experimental or negative control group, while the blank group received 100 μL of high‐sugar DMEM in a 96‐well plate, followed by a 24 h incubation. Post‐incubation, the culture medium was replaced with 200 μL of ethanol solution at varying concentrations (200, 400, 600, 800, 1000, 1200, or 1400 mmol/L) in the experimental group, and incubated for either 4 h or 6 h. Afterwards, 20 μL of 5 mg/mL MTT solution was added and incubated for an additional 4 h. Subsequently, the supernatant was drawn using a syringe, and 150 μL of DMSO was added. Following a 10‐min reaction period, the absorbance at 570 nm was measured in each well using an enzyme marker (Thermo Fisher Scientific, USA). The cell survival rate was then determined using formula (1).

Cell survival (%) = (*A*_2_−*A*_0_)/(*A*_1_−*A*_0_) × 100% Equation (1).

Here, *A*_0_ denotes the OD value of the blank group, *A*_1_ denotes the OD value of the control group, and *A*_2_ denotes the OD value of the experimental group.

### 2.5 Effect of SHP on Ethanol‐Induced LO2 Cell Damage

In the experimental and negative control groups, a cell suspension of 8 × 10^4^ cells/mL was introduced into 96‐well plates, while the blank group was provided with 100 μL of high‐sugar DMEM in lieu of the cell suspension. Following a 24‐h incubation, the experimental group received 100 μL of SHP at various concentrations (20, 40, 60, 80, 100 μg/mL), whereas both the blank and negative control groups were supplemented with 100 μL of high‐sugar DMEM each. After a 12‐h incubation, the culture medium was aspirated using a pipette. Then, 200 μL of 800 mmol/L ethanol was dispensed into the experimental group, while 200 μL of DMEM high glucose medium was administered to both the blank and negative control groups. Following an additional 6‐h incubation, 20 μL of 5 mg/mL MTT solution was introduced and allowed to incubate for 4 h. Post‐incubation, the supernatant was withdrawn using a syringe, and 150 μL of DMSO solution was added to the reaction for 10 min, followed by measuring the absorbance at 570 nm in each well using an enzyme marker. The cell survival rate was then determined according to equation (1).

### 2.6 Effect of SHP on ALT, AST, LDH, and GSH Viability and CAT and MDA Contents After Ethanol‐Induced LO2 Cell Injury

The LO2 cell suspension was seeded into 6 cm sterile culture dishes at a density of 2 × 10^5^ cells/mL, with 5 mL of high‐glucose DMEM added to each dish. Once the cell density reached approximately 80%, drugs (SHP or GSH) were separately added and incubated for 12 h. Following this, the culture medium was aspirated, and 5 mL of 800 mmol/L ethanol was added and incubated for 6 h. ALT, AST, and LDH activities were assessed in cell cultures using ALT, AST, and LDH kits. Intracellular levels of CAT, GSH, and MDA were determined using CAT, GSH, and MDA kits.

### 2.7 Effect of SHP on the ROS Content After Ethanol‐Induced LO2 Cell Injury

LO2 cells were seeded in 96‐well plates at a density of 8 × 10^4^ cells/mL, with 100 μL per well, and cultured for 24 h. Following this incubation period, 100 μL of a specific concentration of drugs (SHP or GSH) was added, and the cells were cultured for an additional 12 h. After removing the culture medium, 200 μL of 800 mmol/L ethanol was added, and the cells were cultured for 6 h. Subsequently, 200 μL of DCFH‐DA was added and incubated for 30 min. The fluorescence intensity of each well was measured using an enzyme marker at an excitation wavelength of 488 nm and an emission wavelength of 525 nm.

### 2.8 Western Blot Analysis

LO2 cells were seeded at a density of 2 × 10^5^ cells/mL and cultured in 10 cm dishes for 24 h. Following treatment with 60 μg/mL SHP for 12 h and 800 mmol/L ethanol for 6 h, the cells were harvested using a spatula and centrifuged to collect the cell pellet. The pellet was then incubated on ice for 15 min after adding RIPA lysis buffer, followed by centrifugation at 4°C and 10,000 r/min for 15 min to obtain the cell lysate. A small amount of the cell lysate supernatant was collected according to the instructions of the BCA protein assay kit to determine the protein concentration and protein concentration. According to the measured protein concentration, all the samples were unified to the same concentration, and in accordance with the proportion of 25 μL 5× protein sampling buffer added to 100 mL of sample, 5× protein sampling buffer was added, the mixture was mixed, and then the mixture was placed in a 95°C metal bath to cool for 5 min so that protein denaturation occurred and the sample served as a protein sample. The protein samples were prepared and then either subjected to immediate electrophoresis or stored frozen at −20°C. Electrophoresis was conducted under the following conditions: 70 V for 30 min followed by 120 V for 1.5–2 h. Subsequently, the membrane was transferred in a membrane transfer tank, rotating at 300 mA for 1 h. The NC membrane was removed and blocked in a rapid containment solution for 20 min at room temperature and then rinsed with TBST. β‐Actin, Nrf2, HO‐1, and GCLC primary antibodies (1:1000) were incubated at 4°C overnight, and all antibodies were diluted with antibody diluent. The following day, the membrane was washed and incubated on a horizontal shaker with secondary antibody (1:1000) for 1 h. The membrane was washed, and the color was developed by ECL. ImageJ software was used to calculate the gray value of each band.

### 2.9 Statistics and Analysis

Each experiment was done three times, and the experimental data were presented as means ± standard deviation (SD). The experimental results were statistically analyzed by IBM SPSS Statistics 26 software, and statistical significance was analyzed by one‐way analysis of variance. GraphPad Prism 8.0.2 Software was used for plotting.

## 3 Result

### 3.1 Establishment of the LO2 Cell Injury Model

**FIGURE 1:**
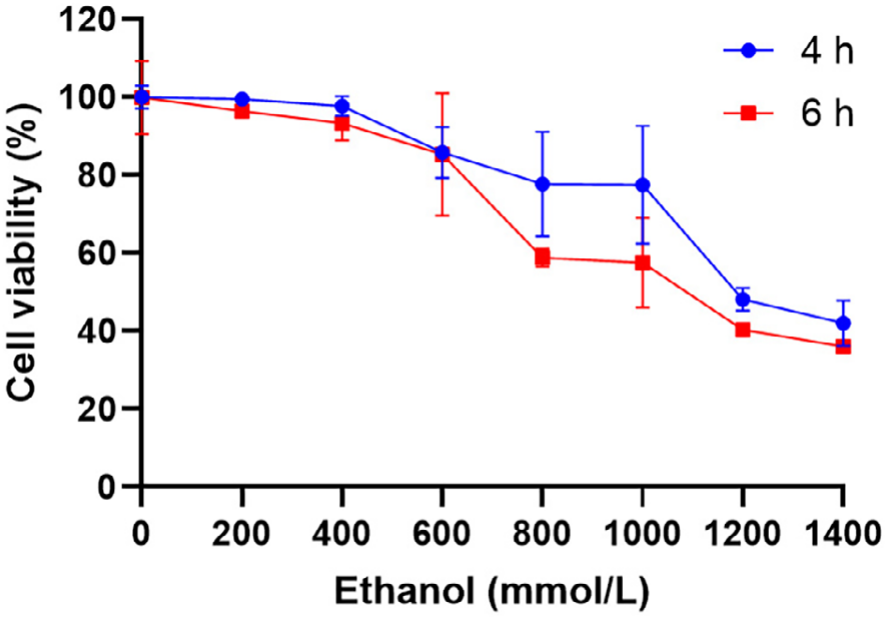
Effects of different concentrations and treatment times of ethanol on cell viability. LO2 cells were treated with ethanol solutions (200–1400 mmol/L) for 4 or 6 h. Then, the cytotoxic effect of ethanol solution on LO2 cells was determined by MTT assay.

As depicted in Figure 1, upon treating the LO2 cells with varying concentrations of ethanol for different durations, a decrease in cell viability was observed with escalating ethanol concentration, with cell viability after 6 h of ethanol exposure being lower than that after 4 h. Specifically, following 6 h of treatment with 800 mmol/L ethanol, the viability of LO2 cells reduced to 51.54% ± 5.11%, indicating moderate damage. Consequently, 800 mmol/L ethanol was selected as the model for inducing ethanol‐induced damage in LO2 cells.

### 3.2 Effect of SHP on Ethanol‐Induced LO2 Cell Viability

**FIGURE 2:**
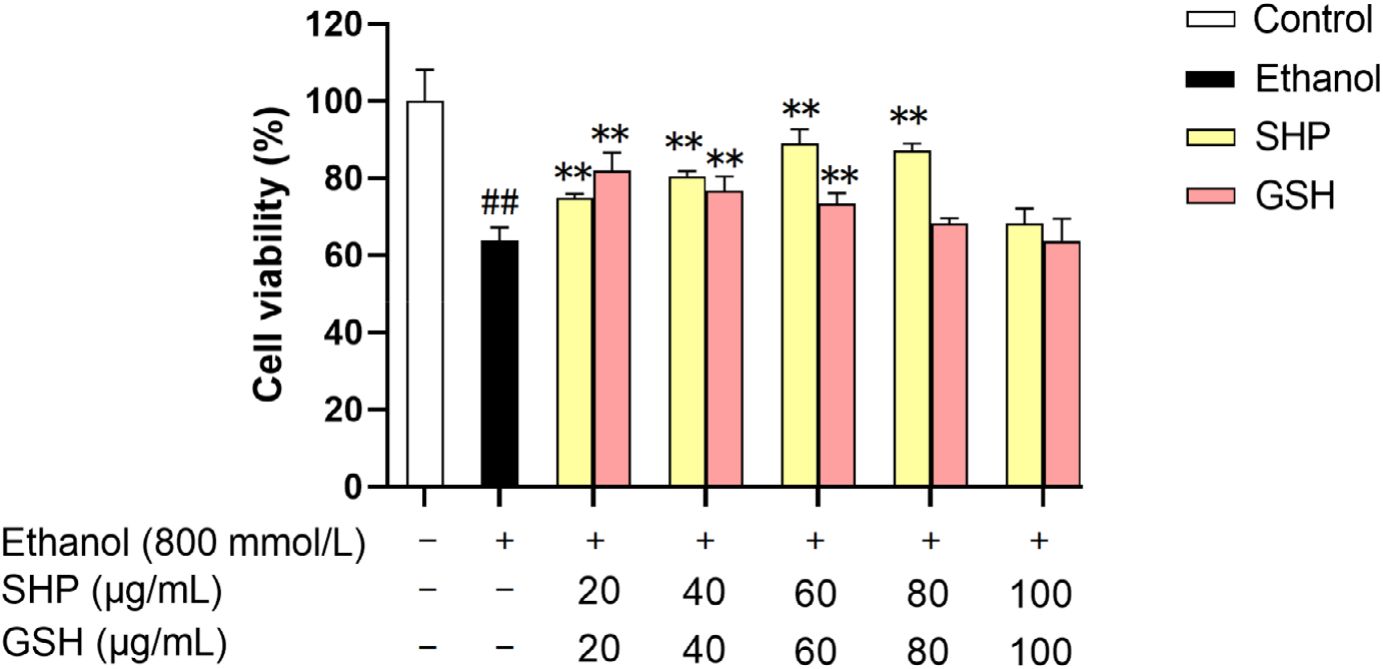
Effects of different concentrations of SHP on ethanol‐induced cell damage. LO2 cells were preincubated with SHP (20–100 μg/mL) for 12 h and then stimulated with ethanol (800 mmol/L ) for 6 h. *p* < 0.01(**) or *p* < 0.05(*) indicates a significant difference between the ethanol and SHP groups. *p* < 0.01 (##) indicates a significant difference between the control and ethanol groups.

As shown in Figure 2, the viability of LO2 cells in the model group was 63.86% ± 3.38% after treatment with 800 mmol/L ethanol for 6 h, indicating that ethanol treatment resulted in cell damage. After pretreatment with different doses of SHP, cell viability increased from 63.86% ± 3.38% to 74.91% ± 1.02%, 80.46% ± 1.36%, 89.17% ± 3.58%, 87.29% ± 1.76%, and 68.24% ± 3.93%, indicating that SHP significantly improved ethanol‐induced hepatocyte injury but did not have a dose‐dependent effect. Moreover, after treatment with 60 μg/mL SHP, cell viability was greatest, indicating that SHP has the best protective effect on ethanol‐induced cell damage. Moreover, the viability of cells treated with 60 μg/mL SHP was greater than that of cells treated with GSH at the same dose, indicating that the protective effect of SHP on ethanol‐induced hepatocyte injury was stronger than that of GSH.

### 3.3 Effects of SHP on ALT and AST Viability After Ethanol‐Induced LO2 Cell Injury

**FIGURE 3:**
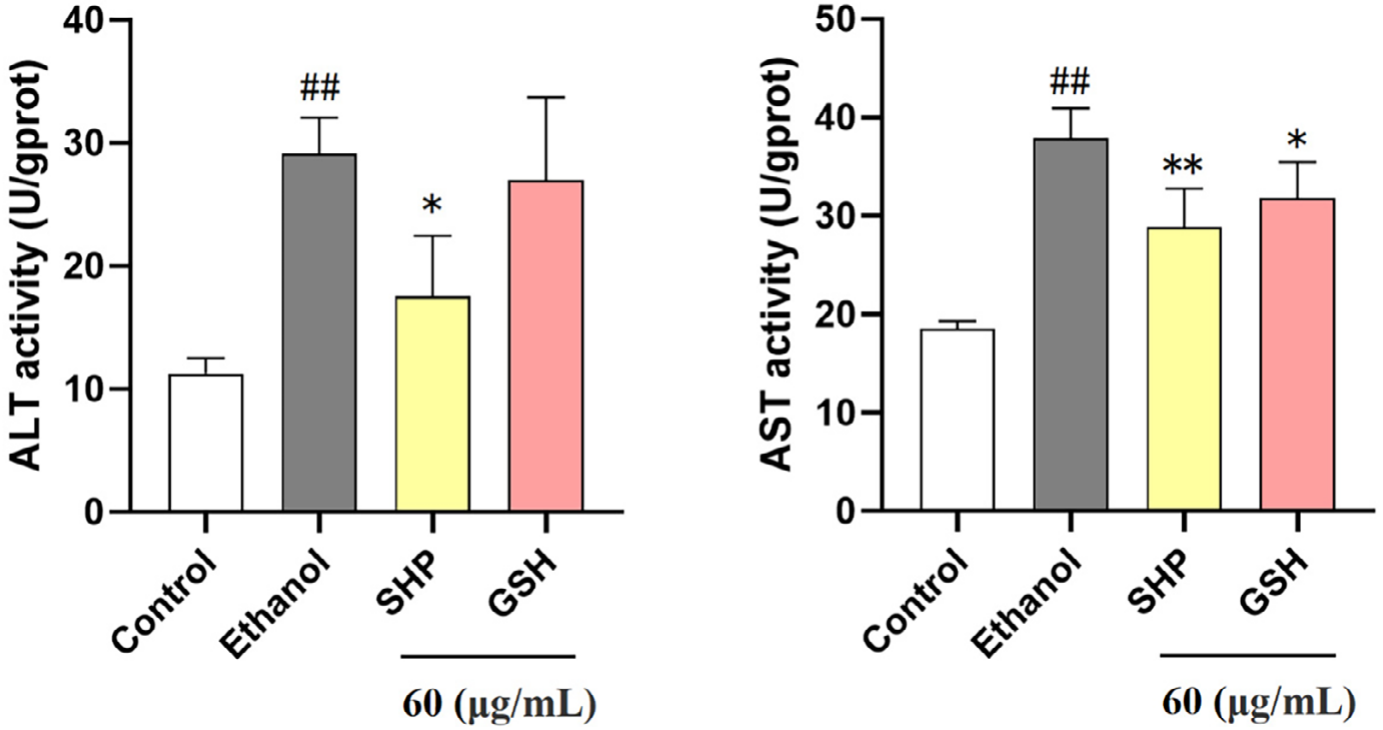
Effects of SHP on ALT and AST activities in ethanol‐treated LO2 cells. LO2 cells were preincubated with SHP and GSH (60 μg/mL) for 12 h and then stimulated with ethanol (800 mmol/L) for 6 h. *p* < 0.01 (**) or *p* < 0.05 (*) indicates a significant difference between the ethanol group and the SHP group. *p* < 0.01 (##) indicates a significant difference between the control group and the ethanol group.

The preceding investigations demonstrated that 60 μg/mL SHP exhibited the most pronounced protective effect against alcohol‐induced LO2 cell injury. Consequently, we selected this concentration for subsequent experiments. ALT and AST activities are well‐established as reliable and sensitive physiological markers of alcoholic hepatocellular injury. ALT and AST are primarily located in the mitochondria of hepatocytes. When the cell membrane is compromised, there is an increase in cell membrane permeability, leading to the rapid release of ALT and AST from hepatocytes into the bloodstream. Hence, the levels of AST and ALT serve as indicators to ascertain hepatocyte damage (Kim et al. 2008; McGill 2016). As shown in Figure 3, ALT and AST viability in the model group was highly significantly greater than that in the negative control group (*p* < 0.01). Compared with those in the model group, the viability of the cells treated with 60 μg/mL SHP decreased from 29.12 ± 2.94 and 37.91 ± 3.01 U/gprot in the model group to 17.53 ± 4.94 and 28.91 ± 3.85 U/gprot, respectively. This may be due to the antioxidant activity of SHP preventing the release of intracellular ALT and AST caused by cell membrane rupture due to the increase in reactive oxygen species (ROS) in the cells, which leads to a corresponding reduction in extracellular ALT and AST activity. Moreover, the ALT and AST levels in the GSH group were higher than those in the SHP group, indicating that the antioxidant activity of SHP was superior to GSH. These findings suggested that SHP could improve liver function to protect LO2 cells damaged by alcohol.

### 3.4 Effects of SHP on LDH Release, CAT and GSH Activities, and MDA Content After Ethanol‐Induced LO2 Cell Injury

**FIGURE 4:**
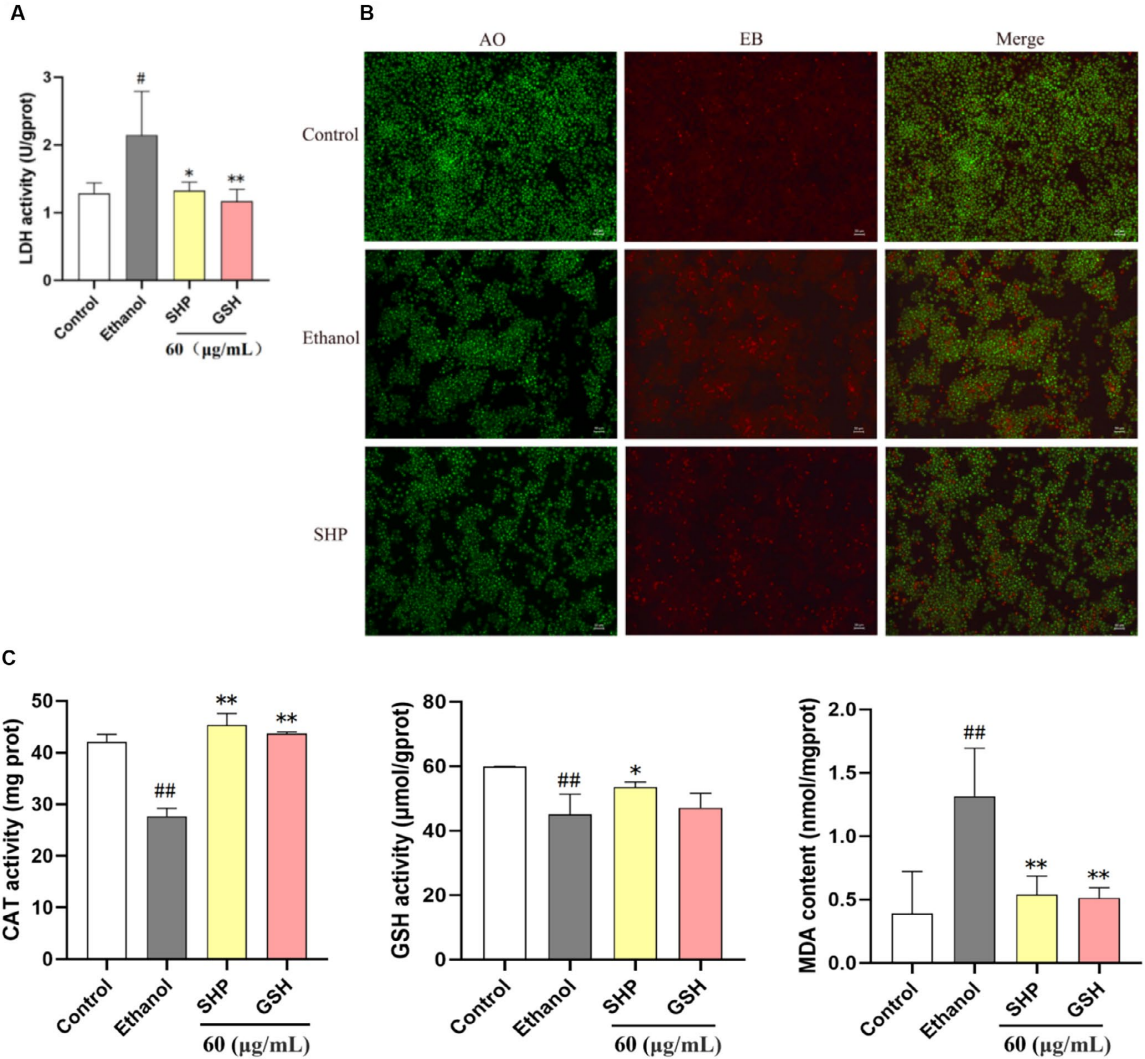
(A) Effect of SHP on LDH release content in ethanol‐treated LO2 cells. (B) Representative images of cell fluorescence indicating cell apoptosis detected by AO/EB double staining (50 μm). The cells were precultured with SHP at a concentration of 60 μg/mL for 12 h and then subjected to ethanol (800 mmol/L) treatment for 6 h. Then, the cells were stained AO/EB solution for 15 min and fluorescence intensity of cells was observed under an inverted microscope. (C) Effects of SHP on CAT activity, GSH activity, and MDA content in ethanol‐treated LO2 cells. LO2 cells were preincubated with SHP or GSH (60 μg/mL) for 12 h and then stimulated with ethanol (800 mmol/L) for 6 h. *p* < 0.01 (**) or *p* < 0.05 (*) indicates a significant difference between the ethanol group and the SHP group. *p* < 0.01 (##) or *p* < 0.05 (*) indicates significant differences between the control group and the ethanol group.

LDH serves as an indicator of cell damage; it is rapidly released from cells upon plasma membrane rupture, which occurs when cells are stimulated to die; thus, it is commonly employed as a marker in cytotoxicity studies (Kumar, Nagarajan, and Uchil 2018). Therefore, an LDH kit was utilized to assess LDH activity in the culture medium. The results, depicted in Figure 4A, show that LDH release in the alcohol‐treated group increased significantly, with extracellular LDH activity rising from 1.28 ± 0.16 (control) to 2.15 ± 0.65. Following treatment with 60 μg/mL SHP, extracellular LDH activity was reduced to 1.33 ± 0.12 U/gprot, a reduction lower than that observed with GSH. This result demonstrated that SHP could protect cells from ethanol‐induced LO2 cell damage. In order to verify the above results, we further used AO/EB double staining assay to detect cell apoptosis. AO, as a green fluorescent dye, can enter into normal cells and stain the whole cell green. EB, as a nuclear dye, cannot pass through normal cells and can only enter into the cell when the permeability of the cell membrane is altered, emitting red fluorescence. As shown in Figure 4B, in the control group, the LO2 cells showed normal morphology and uniform green fluorescence. After ethanol treatment, the LO2 cells contracted and rounded, and some cells entered into late apoptosis (orange) or necrosis (red). However, after SHP pretreatment, the red fluorescence was found to be weak, indicating that SHP could effectively reduce ethanol‐induced cell apoptosis or necrosis, thereby protecting cells from ethanol‐induced cell damage.

CAT is recognized as a key enzyme in alcohol detoxification, aiding in the oxidation of ethanol by converting ethanol to acetaldehyde and H_2_O_2_ to H_2_O (Li et al. 2023). Excessive alcohol consumption can also lead to severe oxidative stress in the liver through the generation of free radicals and increased levels of MDA, which results in hepatocellular damage and apoptosis. MDA, a by‐product of lipid peroxidation, indirectly reflects the extent of free radical damage in the liver (Chen et al. 2016). Conversely, the antioxidant defense system represented by GSH can protect hepatocytes from oxidants, scavenge lipid peroxides, and oxygen free radicals, and shield hepatocytes from ROS damage. GSH levels directly indicate the antioxidant capacity of the liver (Ding et al. 2015). Therefore, to determine whether SHP exerts an antioxidant effect on alcohol‐induced injury in LO2 cells, activities of CAT and GSH, as well as MDA content, were measured. These findings are presented in Figure 4C. Compared with those in the control group, the CAT and GSH levels decreased significantly, and the MDA content increased significantly in the model group. Compared with those in the model group, cells treated with 60 μg/mL SHP exhibited increased CAT and GSH levels and decreased MDA levels, which were greater than those in the GSH group, except for MDA. These results suggest that SHP can improve oxidative stress levels to protect LO2 cells from alcohol‐induced damage.

### 3.5 Effect of SHP on ROS Content After Ethanol‐Induced LO2 Cell Injury

**FIGURE 5:**
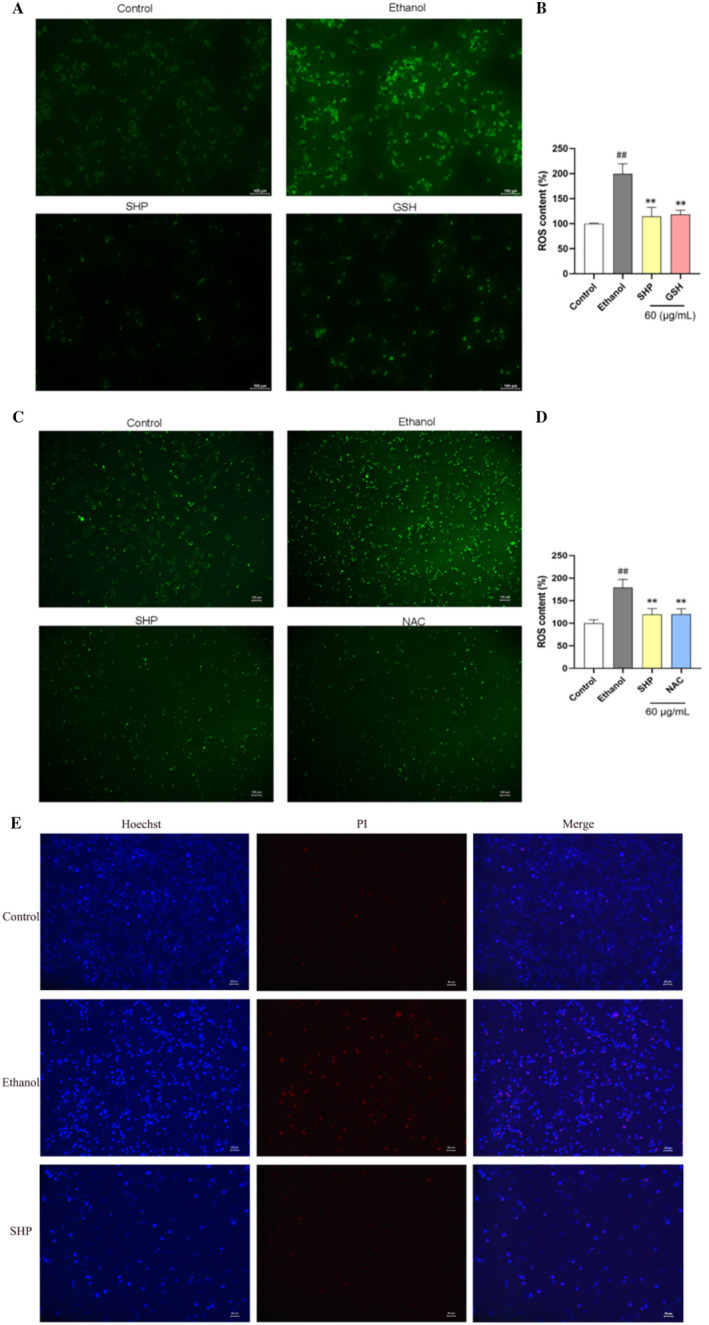
Effect of SHP on reactive oxygen species (ROS) levels in LO2 cells. The cells were precultured with SHP or GSH at a concentration of 60 μg/mL for 12 h and then subjected to alcohol (800 mmol/L) treatment for 6 h. (A) Representative images of cell fluorescence indicating ROS levels detected by DCFH‐DA (100 μm). (B) ROS content in different treatment groups. ROS content was analyzed by measuring the fluorescence intensity of DCFH‐DA. *p* < 0.01 (##) indicates a significant difference between the control and ethanol groups; *p* < 0.01 (**) indicates a significant difference between the ethanol and SHP groups. (C) Representative images of cell fluorescence cells in different treatment groups (100 μm). The cells were precultured with SHP or NAC at a concentration of 60 μg/mL for 12 h and then subjected to alcohol (800 mmol/L) treatment for 6 h. Fluorescence microscopy was used to observe the changes in the fluorescence intensity of LO2 cells in the different groups. (D) Effect of NAC on ROS content. ROS content was analyzed by measuring the fluorescence intensity of DCFH‐DA. *p* < 0.01 (##) indicates a significant difference between the control and ethanol groups; *p* < 0.01 (**) indicates a significant difference between the ethanol and SHP groups. (E) Representative images of cell fluorescence indicating cell death detected by Hoechst 33342 and propidium iodide (PI) double staining (50 μm). The cells were precultured with SHP at a concentration of 60 μg/mL for 12 h and then subjected to ethanol (800 mmol/L) treatment for 6 h. Then, the cells were stained Hoechst33342/PI solution for 20 min and fluorescence intensity of cells was observed under an inverted microscope.

In the development of ALD, alcohol‐induced oxidative stress results in elevated ROS levels in hepatocytes (Chen, Zhong, and Xu 2023). Therefore, we further examined the intracellular ROS levels. As shown in Figure 5A, the ethanol treatment resulted in strong green fluorescence, indicating that ethanol increased ROS levels. However, the green fluorescence in the SHP treatment group was weak, which was weaker than that in the GSH group, indicating that SHP could reduce the increase of ROS content induced by ethanol. Moreover, this result is consistent with that of Figure 5B. Compared with the control group, ROS levels in the model group rose significantly from 100% ± 0.87% to 199.40% ± 20.37%. Treatment with SHP notably reduced the intracellular ROS levels, suggesting that SHP could mitigate ethanol‐induced damage in LO2 cells by lowering ROS levels. This reduction is likely due to the antioxidant properties of SHP, which effectively scavenge free radicals within cells to decrease ROS levels and provide cellular protection. Additionally, ROS levels in the SHP‐treated group were lower than those in the GSH‐treated group (Figure 5B). In order to verify the above results, NAC, a ROS inhibitor was employed to reduce ROS levels. The results showed that ROS levels were reduced to 119.97% ± 12.24% after adding NAC, with no significant difference between the SHP and NAC groups (Figure 5C,D). These data confirm that SHP reduces ethanol‐induced liver injury by alleviating ROS‐induced oxidative stress.

Studies have shown that ROS are central to cell signaling and regulate the major pathways of apoptosis mediated by mitochondria, death receptors, and the endoplasmic reticulum (Moloney and Cotter 2018). Therefore, we further used Hoechst 33342 and propidium iodide (PI) double staining to detect cell death. As shown in Figure 5E, after ethanol treatment, LO2 cells showed strong red and strong blue fluorescence, indicating that ethanol resulted in cell necrosis. However, SHP treatment resulted in weak red fluorescence and strong blue fluorescence observed in LO2 cells, indicating that SHP could improve the fluorescence intensity of the cells in the ethanol‐treated group, and it can be hypothesized that the SHP can effectively prevent cell necrosis caused by ethanol treatment.

### 3.6 Effect of SHP on the Expression Levels of Oxidative Stress‐Related Proteins in Ethanol‐Induced LO2 Cell Injury

**FIGURE 6:**
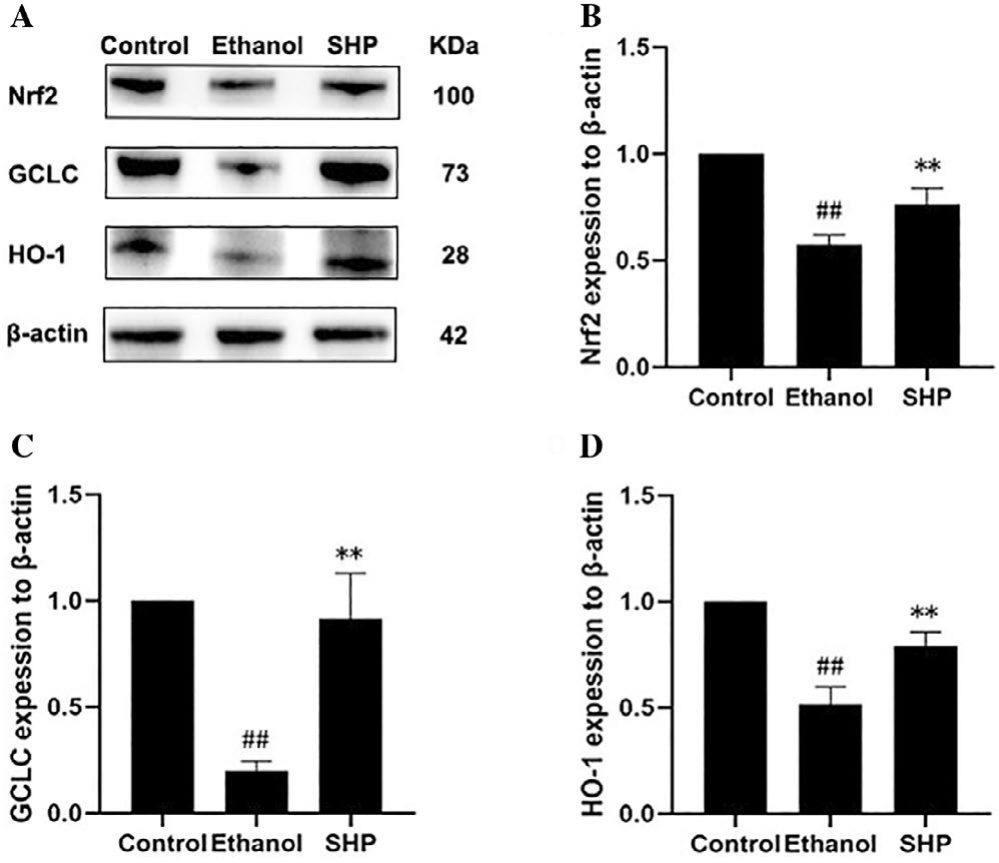
Effect of SHP on the expression levels of oxidative stress‐related proteins in ethanol‐induced LO2 cell injury. LO2 cells were preincubated with SHP (60 μg/mL) for 12 h and then stimulated with ethanol (800 mmol/L) for 6 h. *p* < 0.01 (**) or *p* < 0.05 (*) indicates a significant difference between the ethanol and SHP groups. *p* < 0.01 (##) indicates a significant difference between the control and ethanol groups.

Nrf2 is a transcription factor that plays a crucial role in the regulation of genes encoding antioxidant proteins, detoxification enzymes, metabolic modifiers, and stress‐responsive proteins by binding to enhancer sequences within gene promoters (Keleku‐Lukwete, Suzuki, and Yamamoto 2018; Lu et al. 2016). Activation of the Nrf2 signaling pathway is considered a promising therapeutic approach for treating ALD (Sun et al. 2018). As depicted in Figure 6, compared with the control group, the protein expression levels of Nrf2, HO‐1, and GCLC were significantly reduced in the model group (*p* < 0.05), indicating that alcohol impairs the liver's antioxidant defense system. However, pretreatment with SHP significantly enhanced the protein expression levels of Nrf2, HO‐1, and GCLC in comparison with the model group (*p* < 0.05). This suggests that SHP may activate the Nrf2/HO‐1 signaling pathway and upregulate the activity of the downstream antioxidant enzyme GSH, which contributes to restore the balance of the liver's antioxidant system, thereby protecting the liver from alcohol‐induced peroxidative damage.

## 4 Discussion

Fucoidan shows considerable potential in the treatment of liver diseases due to its antioxidant, immune‐enhancing, antiviral, and antitumor properties. However, the complexity of its molecular structure results in variations in the composition and positioning of sulfate groups across different sources of fucoidan. The relationship between its structural composition and its activity remains unclear, making this an area deserving of thorough research and significant attention.

Alcoholic liver disease is among the most prevalent causes of liver injury worldwide. The specific pathogenic mechanisms through which the polysaccharide from *Sargassum* protects against alcoholic liver injury remain inconclusive. It is hypothesized that alcohol metabolites, oxidative stress, and inflammatory mediators contribute to alcoholic liver injury. The proposed mechanisms include oxidative stress, inflammatory response, endoplasmic reticulum stress, immune response, and lipotoxic metabolic injury (Louvet and Mathurin 2015). Chronic or excessive alcohol consumption can inhibit the oxidation of acetaldehyde in the mitochondria, leading to the continuous accumulation of acetaldehyde, an alcohol metabolite, in the liver. This accumulation can disrupt the inner membrane structure of hepatocytes, damage mitochondria, affect the microtubular system of the liver, and induce hepatic fibrosis, thereby causing liver injury. Additionally, excessive alcohol undergoes oxidative reactions in the liver, generating a high concentration of reactive oxygen species and significantly reducing the levels of antioxidant substances such as glutathione. This leads to the degradation of mitochondrial structure and ultimately results in liver injury (Ding et al. 2015; Louvet and Mathurin 2015).

In this study, the human hepatocyte cell line LO2 was utilized to establish a cellular model of alcoholic liver injury. The levels of ALT, AST, and LDH in the culture medium of LO2 cells after ethanol injury were extremely significantly elevated (*p* < 0.01), which indicated that ethanol damaged the cell membrane structure of LO2 cells to a certain extent. After treatment with SHP, the viability of LO2 cells cultured with ALT, AST, and LDH was reduced, which indicated that SHP could protect the cell membrane structure of LO2 cells. After ethanol damage, the intracellular GSH viability decreased significantly (*p* < 0.01), and the MDA content increased significantly (*p* < 0.01), which indicated that ethanol disrupted intracellular redox homeostasis and damaged the cells. When LO2 cells were treated with SHP, a significant restoration of intracellular GSH activity and a notable reduction in MDA content were observed, suggesting that SHP effectively mitigates ethanol‐induced cellular damage. Additionally, ethanol undergoes oxidation by cytochrome P450 (CYP2E1) in the liver, a process that generates excessive reactive oxygen species (ROS), key contributors to intracellular oxidative stress. An increase in ROS levels in hepatocytes, stimulated by excess ethanol, leads to liver injury (Liu et al. 2015; Pan et al. 2016). Treatment with SHP in ethanol‐induced cells showed a reduction in intracellular ROS content, indicating that SHP can inhibit the ethanol‐induced oxidative stress within cells, thereby providing cell protection. These findings align with those reported by Chen et al., whose study showed that CUR/CDP ameliorates ethanol‐induced LO2 cell injury by enhancing the activity of associated antioxidant enzymes and reducing intracellular ROS levels (Chen et al. 2022). Furthermore, Lim et al. reported that fucoidan from *Murraya* spp. prevented alcohol‐induced liver injury by modulating inflammatory mediators in mice and HepG2 cells, suggesting a novel therapeutic approach (Lim et al. 2015).

In alcoholic liver disease, oxidative stress in hepatocytes is an important pathogenic mechanism. Free radicals and reactive oxygen species produced during alcohol metabolism lead to oxidative stress, which in turn triggers hepatocellular injury. The Nrf2/HO‐1 signaling pathway may represent a promising target for intervention, as its activation could mitigate the onset and progression of alcoholic liver disease. Studies have reported that the alcoholic extract of five‐fingered walnut has a protective effect on mice with alcoholic liver injury, and the mechanism may be through the activation of Nrf2, which induces the modulation of downstream antioxidant factors or antioxidant systems and inhibits the abnormal activation of CYP2E1 to attenuate the oxidative stress response, thus reducing the damage of alcohol to the liver (Ru, Zhongyuan, and Juan 2021). POP has been shown to alleviate alcohol‐induced oxidative stress in HepG2 cells by regulating the Nrf2/Keap1 pathway, reducing inflammatory indices and apoptosis levels in these cells (Qi et al. 2022).

**FIGURE 7:**
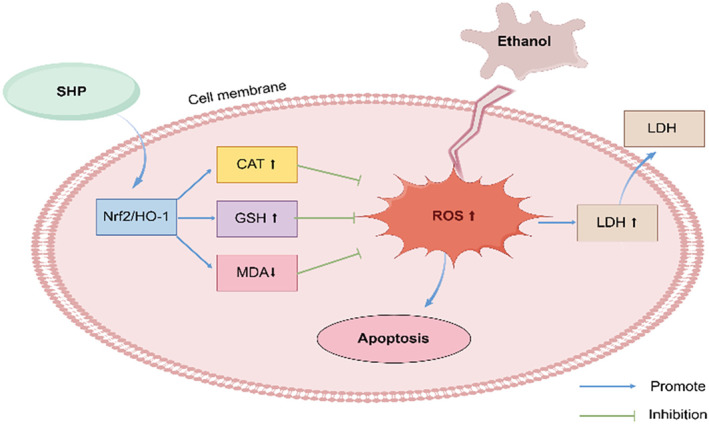
Diagram of the possible signaling pathway of SHP that protects against ethanol‐induced LO2 cell injury.

In this study, the effect of SHP on the activation of Nrf2/HO‐1 signaling pathway was investigated by detecting Nrf2 and HO‐1 levels. The results showed that pretreatment with SHP significantly increased the protein expression levels of cellular Nrf2, HO‐1, and GCLC compared with ethanol ‐treated cells. This suggests that the activation of Nrf2/HO‐1 pathway may be one of the potential pathways for the cytoprotective effect of SHP against alcoholic liver injury. SHP can maintain the balance of the body's antioxidant system by activating the expression of molecules related to the key antioxidant signaling pathway Nrf2/HO‐1 and upregulating the levels of its downstream antioxidant substances, GSH and CAT, downward revision of MDA levels. This contributes to protecting cells from ethanol‐induced cell injury, by FigDraw (Figure 7).

## 5 Conclusion

In this study, we established an ethanol‐induced hepatocyte injury model and utilized biochemical kits to assess the impact of SHP on the activities of these enzymes following ethanol‐induced cell injury. The results from in vitro experiments indicated that SHP exhibits a protective effect against alcoholic hepatocellular injury by enhancing the antioxidant capacity of LO2 cells and suppressing lipid peroxidation. The mechanism may be related to the ability of SHP to activate the Nrf2/HO‐1 signaling pathway to protect LO2 cells from alcohol‐induced oxidative damage. In summary, SHP demonstrates the potential to mitigate ethanol‐induced injury in LO2 cells, offering a theoretical basis for its prospective application in liver injury management and for further exploration of its hepatoprotective properties.

## Author Contributions

**Yuxuan Liang:** writing – review and editing, writing – original draft, methodology, formal analysis, data curation, conceptualization. **Zhuo Wang:** writing – review and editing, supervision. **Rui Li:** software, methodology. **Saiyi Zhong:** software, methodology. **Xiaofei Liu:** validation, software, methodology. **Jianping Chen:** validation, formal analysis, project administration, Funding acquisition.

## Conflicts of Interest

The authors declare no conflicts of interest.

## Data Availability

Data sharing is not applicable to this article as no new data were created or analyzed in this study.
